# Supplementary material for: 3D Cultures of Salivary Gland Cells in Native or Gelled Egg Yolk Plasma, Combined with Egg White and 3D-Printing of Gelled Egg Yolk Plasma
Source: Materials (Basel). 2019 Oct 24;12(21):3480. doi: 10.3390/ma12213480 (PMC6861896; doi:10.3390/ma12213480)
Supplement: Supplementary file 1 [file materials-12-03480-s001.zip › materials-598611-SI.pdf]

Article

3D Cultures of Salivary Gland Cells in Native or Gelled Egg Yolk Plasma Combined with Egg White and 3D-Printing of Gelled Egg Yolk Plasma

André M Charbonneau [1], Joseph M Kinsella [2] and Simon D Tran [1,*]

[1] Faculty of Dentistry, Craniofacial Tissue Engineering and Stem Cells Laboratory, McGill University, H3A 0C7 Montréal, QC, Canada
[2] Faculty of Engineering, Department of Bioengineering, McGill University, H3A 0C7 Montréal, QC, Canada
* Correspondence: simon.tran@mcgill.ca

Received: 4 September 2019; Accepted: 16 October 2019; Published: 24 October 2019

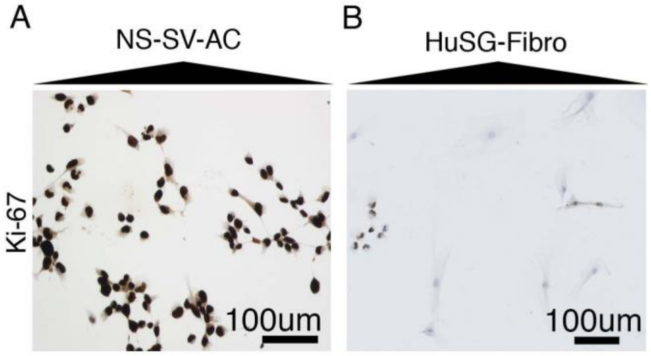

Figure S1. Characteristics of NS-SV-AC and HuSGFibro grown with cell culture media on glass. We seeded the cells grown on glass with media and in a humid glass chamber, and incubated the glass chamber with glass slides for four days. The samples were then removed and fixed with 10% neutral buffered saline and cell permeated with 1% Triton× 100. (**A**) The results for the NS-SV-AC; (**B**) The results for the HusSG-Fibro. For immunohistochestry, a brown colour is indicative of the presence of protein. Panel A reproduced with permission from our own publication in Wiley's Biotechnology Journal. The Charbonneau et al. article was titled "3D Culture Histology Cryosectioned Well Insert Technology Preserves the Structural Relationship between Cells and Biomaterials for Time-Lapse Analysis of 3D cultures" © 2019 WILEY-VCH Verlag GmbH & Co. KGaA, Weinheim.

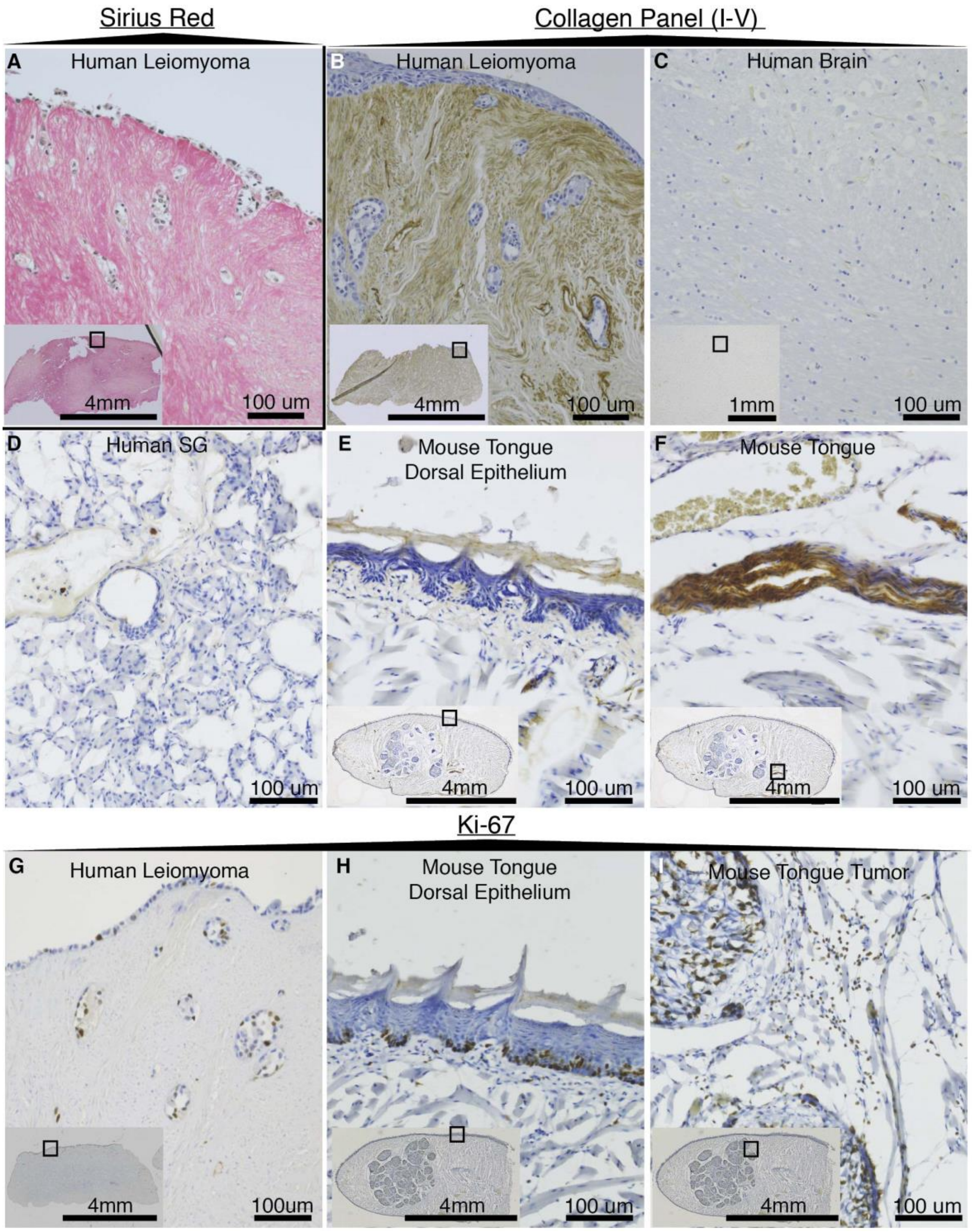

Figure S2. Results obtained from some tissues stained as controls. All human leiomyomas (**A,C,G**) were pieces of leioyoma tissues overlaid with cancer cells. All mouse tongues (**E,F,H,I**) had tumours. Low magnifications at 25× are found on bottom left and within show a black square signifying the location of the higher magnification 200× image (**A,B**) Tissue stained with Sirius Red. Most often, red signify collagen, while nuclei are black. (**B,C,D,E,F**) Tissues stained with anti-Collagen Panel (I-V) antibody by IHC and DAB. Collagens I-V are various shades of brown, while nuclei are pale blue. (**F,G,H**) Tissues stained with anti-Ki-67 antibodies. Ki-67 positive cells have dark brown nuclei stained by IHC and DAB, while non-proliferating nuclei are pale blue. Panels (**A,B,C**) reproduced with permission from our own publication in Wiley's Biotechnology Journal. The Charbonneau et al. article was titled "3D Culture Histology Cryosectioned Well Insert Technology Preserves the Structural Relationship between Cells and Biomaterials for Time-Lapse Analysis of 3D cultures" © 2019 WILEY-VCH Verlag GmbH & Co. KGaA, Weinheim.

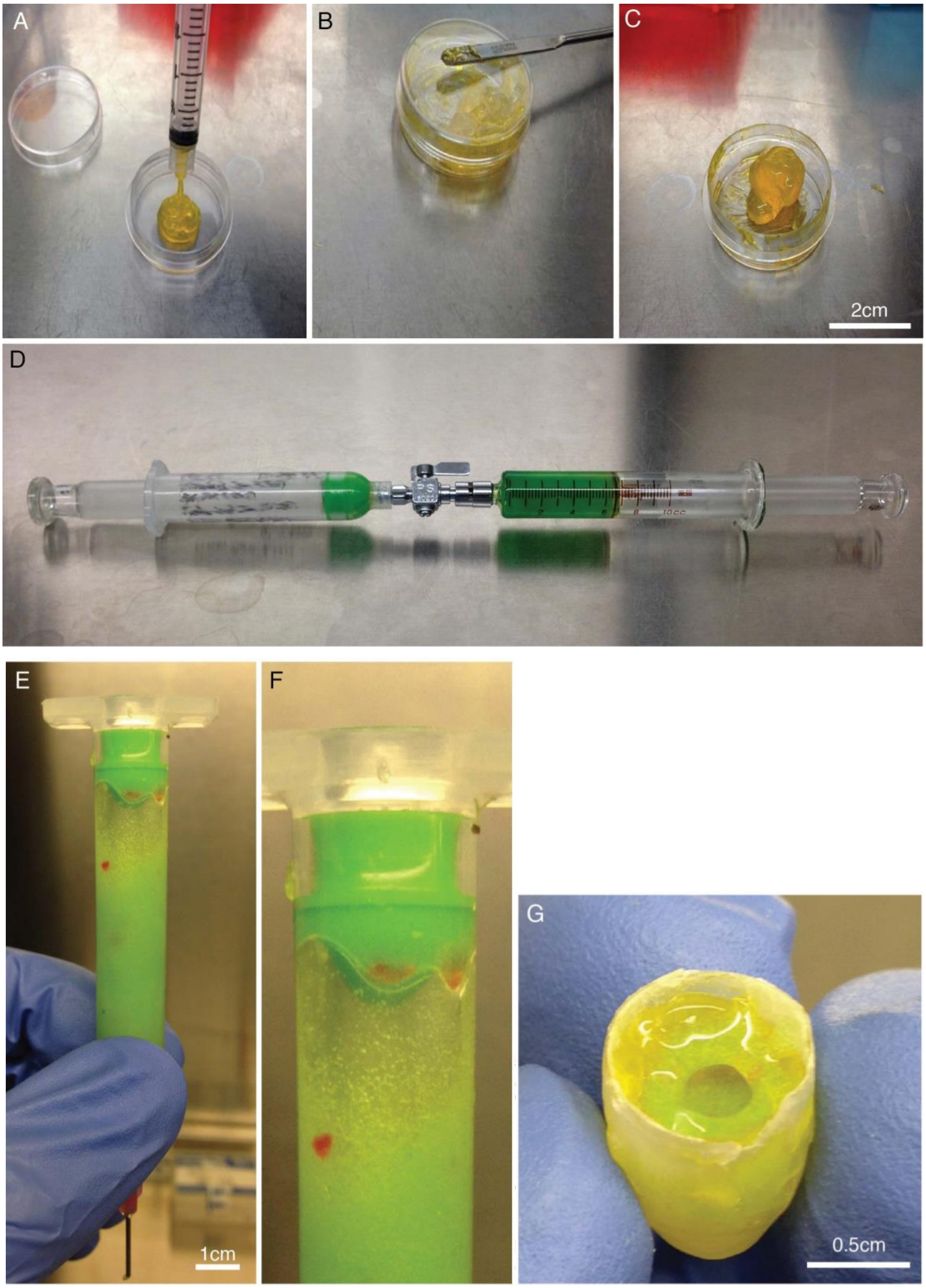

Figure S3. The methods used to mix cells in gEYP and observations. (**A,B,C**) Manual mixing in a Petri dish with a metal spatula (**A**) Extrusion of GEYP into a dish; (**B**) Mixing with the spatula; (**C**) Clump of GEYP in a dish; (**D,E,F**) Mixing by connected syringes and stopcock; (**D**) GEYP with green food colouring containing cells mechanically mixed. A glass and plastic syringe were connected with a metal stopcock and piston action of both syringes mixed the food colouring and cells into the gEYP; (**E,F**) Resulting appearance after mixing. Digital zoom in F show bubble formation; (**G**) Gel's appearance after piston mixing and extrusion. Still a gel, but weaker than before the mix.

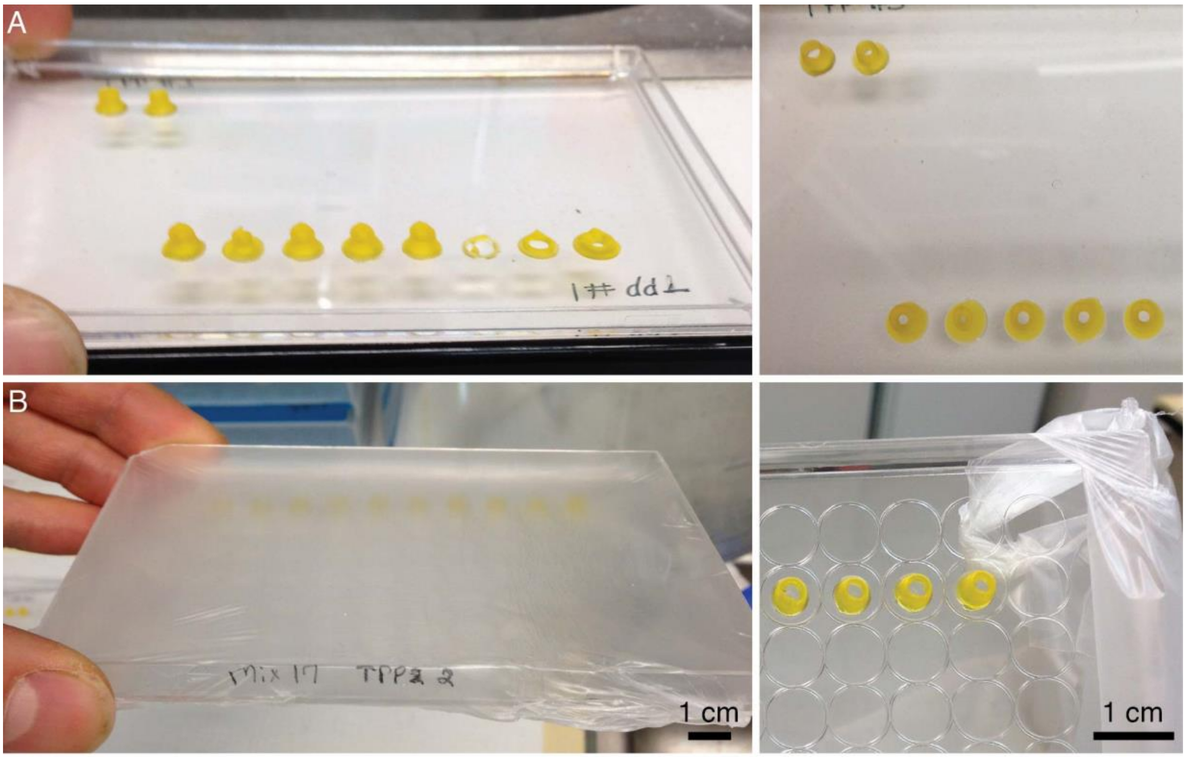

Figure S4. Appearance of gEYP several days after printing. (**A**) No paraffin paper seal; (**B**) with paraffin paper seal.

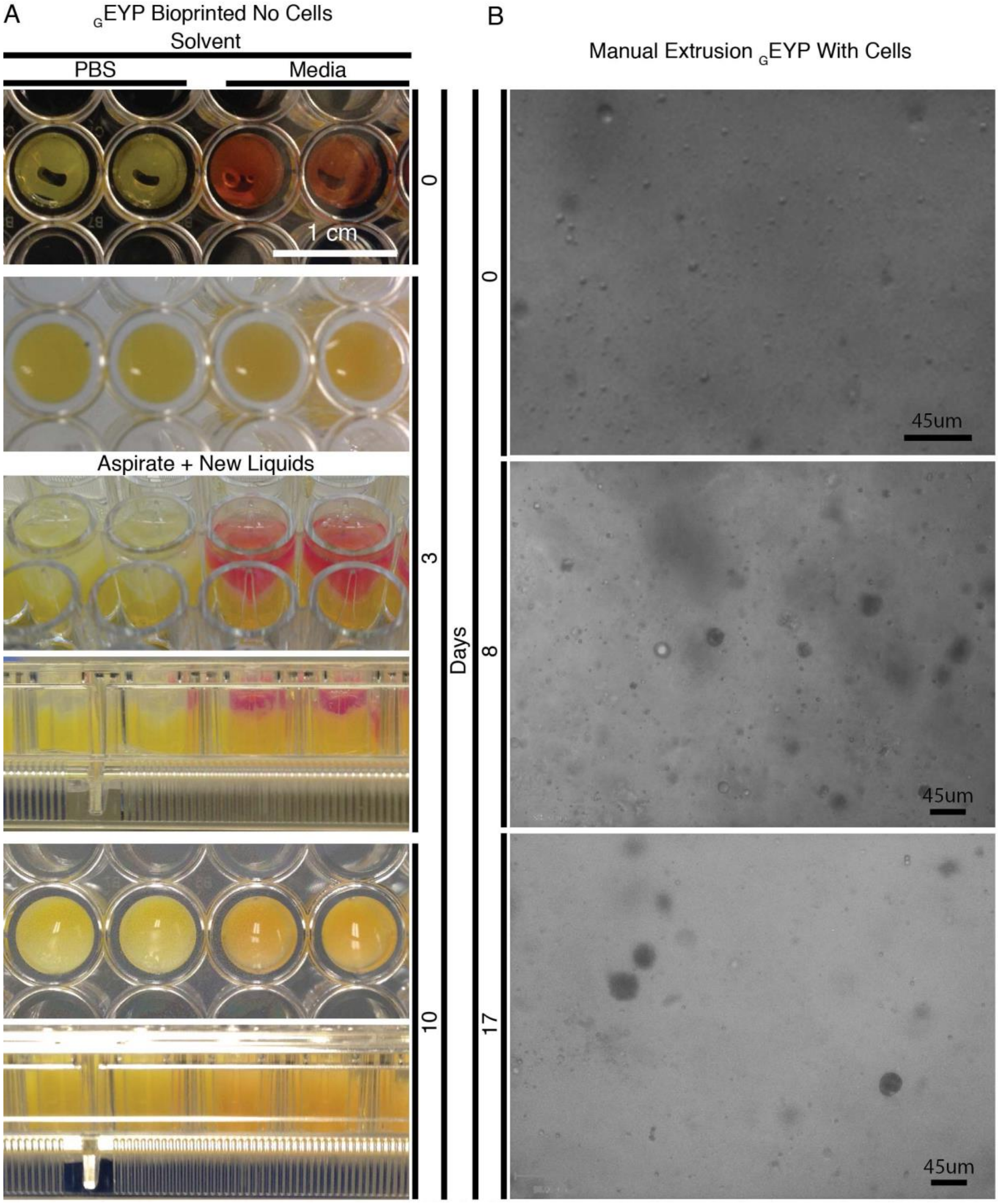

Figure S5. GEYP behaviour with and without cells when exposed to PBS and cell culture media. (**A**) We 3D-printed the shape of a cylinder into a 96 well plate to evaluate how PBS and cell culture media degrade GEYP. PBS and media were added to the cylinder After 3 days, we could still aspirate the media and the relative structure of the cylinder could be seen. At 10 days, when aspirating the liquid, the gelled structured could not be distinguished from the liquid; (**B**) we manually extruded cells in GEYP to evaluate how cells embedded in the biomaterial are distributed over days (i.e. if they sink or remain in 3D). Black and white images of cells in GEYP over 17 days. First, a master mix of GEYP and cells was made in a 30-mm dish by manually mixing the cells in the gel with a metal spatula. Master mix was calculated to yield in each well~200 µL of GEYP and~15,000 cells. Master mix of GEYP with cells was transferred to a 10 cc syringe, then 1 cc syringe, and extruded through an 18 g needle. Every 4 days, we added 40 µL media/well. Over the entire experiment, GEYP maintained 3D-distribution of the cells in certain sections of each well.

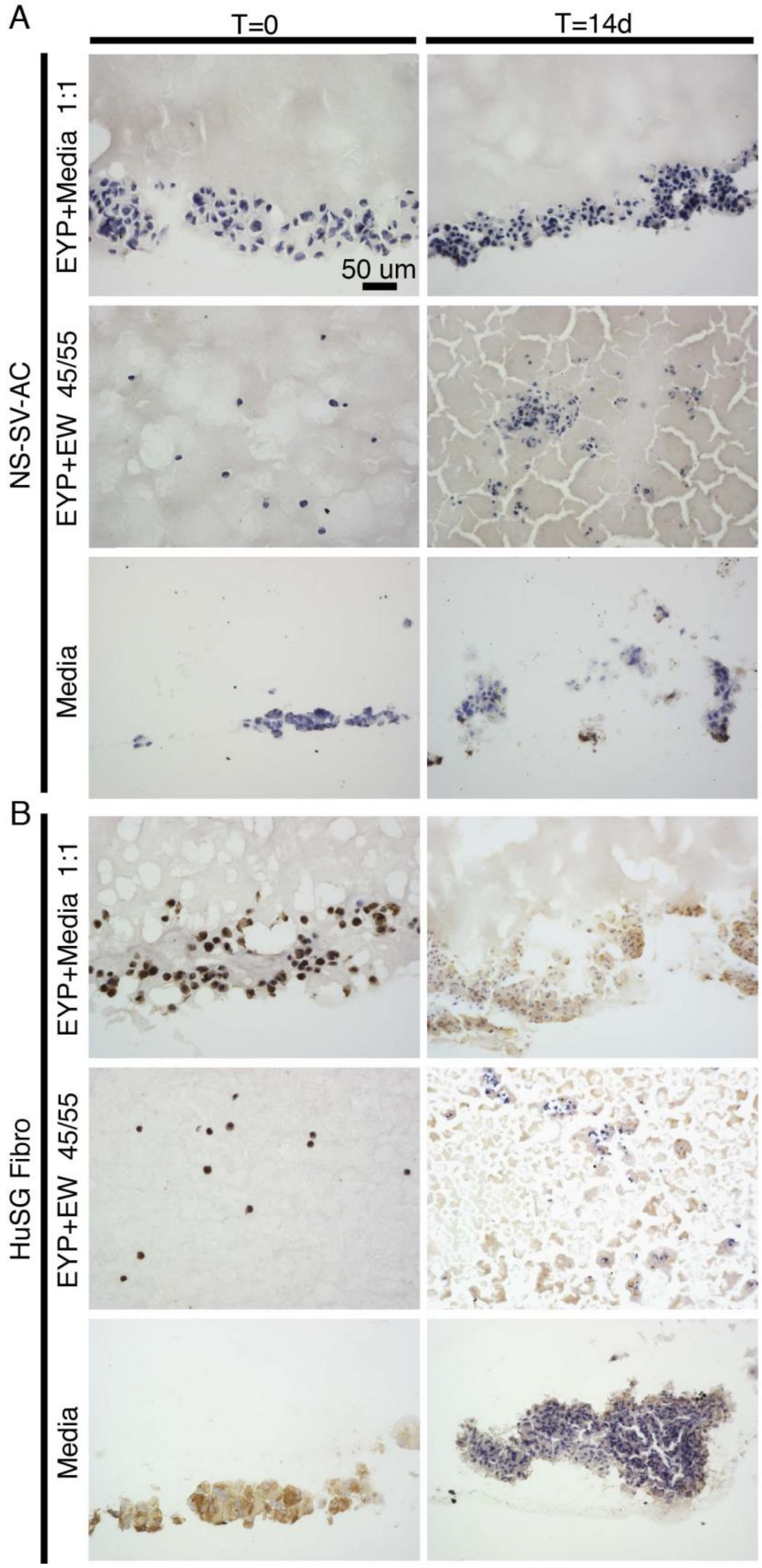

Figure S6. NS-SV-AC and HuSG Fibro cells in EYP biomaterial combinations and in cell culture media analyzed at 0 and 14 Days with anti-collagen panel antibody using immunohistochemistry on slides obtained from sectioning 3D-Cryo Well Inserts. Images were captured with 20× objective. Biomaterials commonly appear pale grey, while nuclei are pale blue. Presence of collagen was shown in dark brown. (**A**) NS-SV-AC cells in the biomaterials and media; (**B**) HuSG Fibro cells in the biomaterials and media. Materials and Methods: The Collagen Panel (collagen I through V) antibody

(PA136058, ThermoFisher) was diluted in antibody diluent at 1:400 (S2022, Dako) and proceeded to immunostaining using autostainer (El72566, LV-l, Labvision). The primary Col Pan antibody was applied for 60 mm and rinsed with 1× TBST. Then, Dako REAL Peroxidase Blocking Solution was added for 5 min and rinsed for 5 min with water and 1X TBST. Afterwards, secondary Bright Vision poly HRP-An-ti-Rabbit IgG (VWRKDPVR500HRP, ImmunoLogic) was used for 30 min, followed by 1× TBST rinse. The DAB + chromogen in substrate buffer (K5007, Dako) was applied for 5 min, followed by l× TBST and water rinse. The Mayers Hema-toxylin HTX Plus (01825, Histolab) was added for 10 min and rinsed with TBST l×. For collagen antibody, a blocking step was tested but did not show difference from no blocking. NS-SV-AC's T = 0 and T = 14 d Collagen Panel in media images were reproduced with Wiley's Biotechnology Journal's permission from our own article. The Charbonneau et al. article was titled "3D Culture Histology Cryosectioned Well Insert Technology Preserves the Structural Relationship between Cells and Biomaterials for Time-Lapse Analysis of 3D cultures" © 2019 WILEY-VCH Verlag GmbH & Co. KGaA, Weinheim.

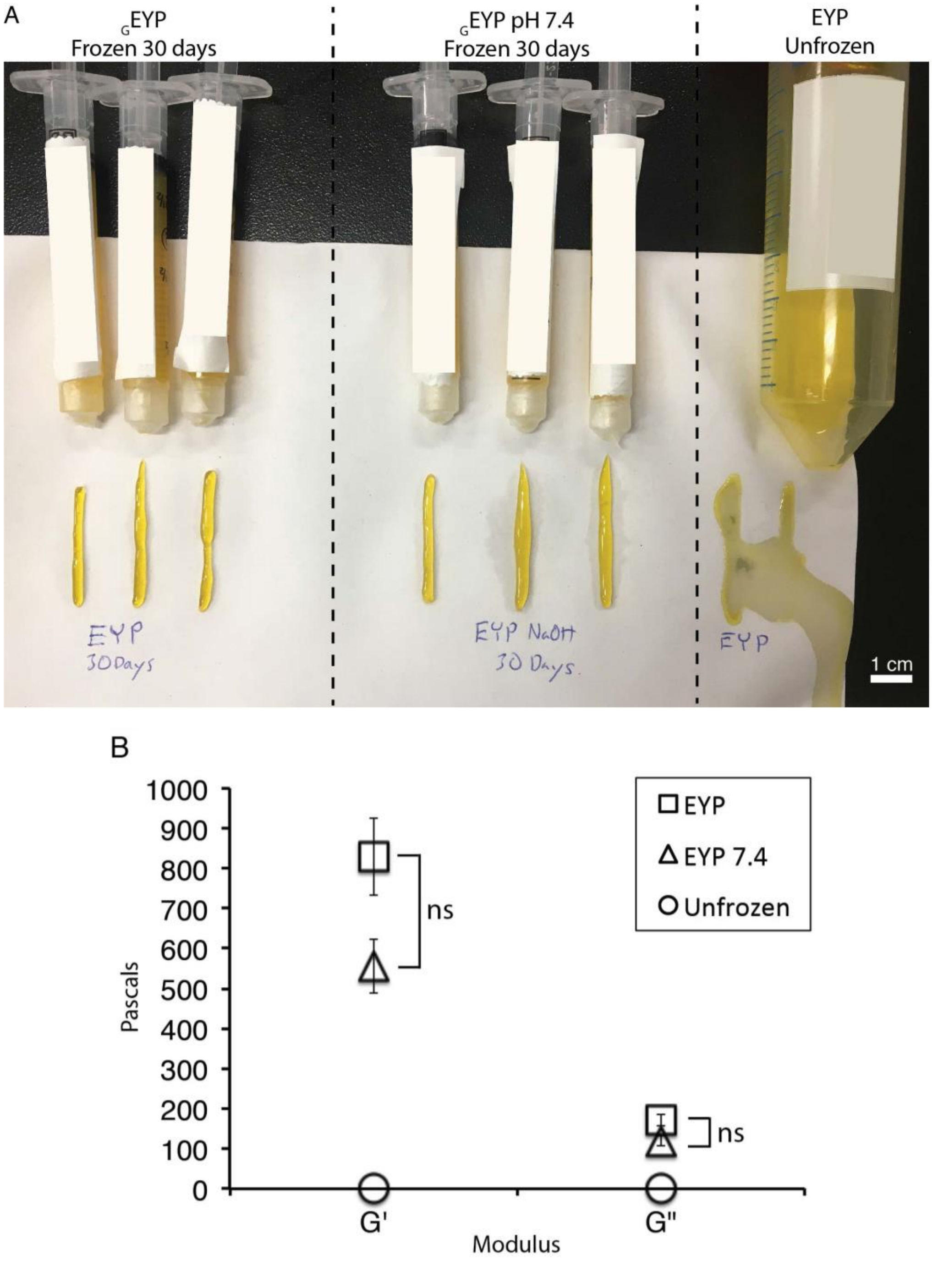

Figure 7. Tests conducted to demonstrate the possibility of creating GEYP with pH-modified EYP. EYP's pH was modified with NaOH to 7.4 (EYP 7.4). (**A**) EYP and EYP 7.4 were placed in syringes and incubated for 30 days at −20 °C. After incubation, we thawed samples to 37 °C and manually extruded the gels on white paper. Gelation appears similar between GEYP and GEYP 7.4; (**B**) Quantification of visco-elastic properties between GEYP, GEYP 7.4, and EYP using a rotational rheometer. Samples were at 37 °C over 15 min using a time test with 0.1 Hz and 0.1% strain. G': Storage Modulus, G" Loss Modulus. Two tailed Student's T test revealed no significant difference between EYP and EYP 7.4. n = 3 as shown in panel A.

© 2019 by the authors. Submitted for possible open access publication under the terms and conditions of the Creative Commons Attribution (CC BY) license (http://creativecommons.org/licenses/by/4.0/).
